# Une fistule recto-vaginale rentrant dans le cadre d'un syndrome de Currarino

**Published:** 2011-12-02

**Authors:** Mounia Lakhdar Idrissi, Abdeladim Babakhoya, Youssef Bouabdellah, Mostapha Hida

**Affiliations:** 1 Unité de gastro-entérologie pédiatrique, service de Pédiatrie, hôpital mère-enfant, CHU Hassan II de Fès, Maroc; 2 Service de chirurgie pédiatrique, hôpital mère-enfant, CHU Hassan II de Fès, Maroc

**Keywords:** Fistule recto-vaginale, agénésie sacrée, masse pré-sacrée, enfant

## Abstract

Le syndrome de Currarino (SC) est défini par une triade rassemblant une malformation ano-rectale, une agénésie sacrée et une tumeur pré-sacrée. Nous rapportons le cas d'une fille de 4 ans et demi ayant été admise en consultation de gastro-entérologie pédiatrique pour constipation avec issue de selle à travers un orifice vulvaire. La radiographie du rachis avait montré une agénésie sacrée. Le fistulo-scanner a mis en évidence une fistule recto-vaginale et l'IRM pelvienne a confirmé l'agénésie sacrée et a retrouvé une méningocèle antérieure. La découverte d'une malformation ano-rectale doit faire chercher une autre anomalie de la triade de Currarino. Cette affection, rare, nécessite une prise en charge médico-chirurgicale assez complexe.

## Introduction

En 1981, Currarino et coll ont identifié l'association de malformations congénitales de l'extrémité caudale regroupant une malformation ano-rectale, une anomalie sacrée et une tumeur pré-sacrée en tant que complexe malformatif. La découverte d'un élément de la triade doit faire rechercher systématiquement les autres composantes de celle-ci. Des anomalies de l'appareil uro-génital peuvent être associées.

## Observation

**Figure 1:**
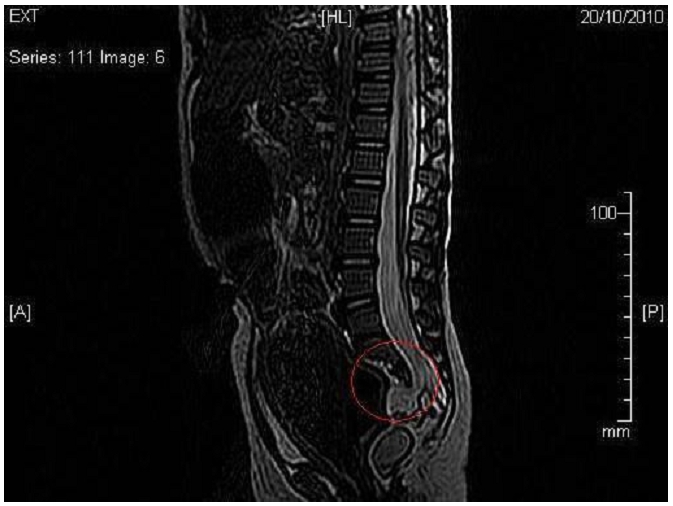
Agénésie sacrée et méningocèle antérieure sur l'IRM médullaire chez un patient présentant une fistule recto-vaginale rentrant dans le cadre d'un syndrome de Currarino

E.N est une fillette de 4 ans et demi qui s'est présentée en consultation de gastro-entérologie pédiatrique pour une constipation chronique et encoprésie. Sa mère est une primipare âgée de 26 ans et son père est de 30 ans; ils ne sont pas consanguins. Sa grossesse était bien suivie avec des sérologies négatives. On avait noté la notion d'oligoamnios sévère sans qu'aucune anomalie prénatale ne soit identifiée. L'accouchement s'est déroulé par voie haute à terme. Le poids de naissance était de 3kg et on avait noté une bonne adaptation à la vie extra-utérine. A la naissance, la fille avait une imperforation anale avec issu de selles à travers un orifice vulvaire antérieur. Elle était opérée à l’âge de 4 mois ou elle a bénéficié en même temps de la fermeture d'une fistule recto-vaginale. A ce moment-là, sur une radiographie standard, on notait une agénésie des 3 vertèbres sacrés et du coccyx. La recherche de la troisième composante du SC n'a pas été menée. Quatre ans plus tard, elle consulte pour la même symptomatologie. La maman rapporte la notion de constipation et toujours l’émission de selles à travers l'orifice vaginal. Nous avons réalisé un fistulo-scanner qui a confirmé la reperméabilisation de la fistule recto-vaginale basse. En parallèle, et devant l'association de la malformation ano-rectale et l'anomalie sacrée, une IRM pelvienne a été demandée. Cet examen a permis de confirmer l'agénésie du sacrum et de mettre en évidence une masse pré-sacrée et retro-rectale ovalaire de 26 mm de diamètre et qui correspond à une méningocèle antérieure (Figure 1). Le diagnostic du syndrome de Currarino a été retenu. Par ailleurs, cette petite n'avait pas d'anomalies urinaires et son examen neurologique était sans particularité. La prise en charge de cette malade exige une reprise en chirurgie pédiatrique ou une colostomie et une ablation de la masse pré-sacrée seront d'abord réalisées.

## Discussion

La triade de Currarino décrite en 1981 par le radiologue américain Guido Currarino associe une malformation ano-rectale, une anomalie sacrée et une tumeur pré-sacrée. C'est une affection autosomique dominante liée à une mutation du gène HLXB9 au locus 7q36. Environ 50% à 60% des cas signalés ont des antécédents familiaux d′anomalies associées à la triade. Aucune corrélation génotype phénotype n'a été mise en évidence [1–2].

L′anomalie embryologique commune de cette triade a été introduite par Currarino et est retenue par plusieurs auteurs, du fait de l′atteinte concomitante; vertébrale, intestinale et neurale au même niveau. La persistance d′une adhésion anormale entre l′endoderme (futur tube digestif) et le neurectoderme (futur tube neural) empêcherait la fusion antérieure du corps vertébral; il en résulterait une « fistule » entre les éléments digestifs d'une part, et neuraux d′autre part. La résorption partielle de cette « fistule » du côté dorsale donnerait une méningocèle et, du côté ventral, un kyste entérique [1].

Le diagnostic prénatal de la triade de Currarino au moyen de l′échographie obstétricale est possible. Il facilite la reconnaissance précoce et la gestion chirurgicale de fistules myélo-rectales persistantes, évitant ainsi la survenue des méningites bactériennes potentiellement mortelles. Une approche thérapeutique anténatale, guidée par ultrasons est discutée [2].

Autrement, le SC peut se révéler par une constipation chronique. En effet, la constipation de longue durée devrait être conçue comme une présentation possible et peut être la seule de ce syndrome [3]. Saberi et al rapporte le cas d'une jeune femme ayant un SC interprété à tord comme maladie de Hirschsprung (MH) pendant 17 ans [4]. La MH peut, cependant, être associée au SC. Dans la série de G Martucciello, cette maladie et d'autres dysganglionoses étaient retrouvées dans 50% des cas. [5]

La survenue des méningites purulentes récidivantes à germes variés (*Escherichia coli*, *Streptocoque B*, *Hæmophilus influenzae*…) peut être révélatrice du SC après avoir éliminé les causes classiques [6].

Les malformations anorectales décrites dans le SC sont rarement une fistule recto-vaginale, comme le cas de notre observation. Il s'agit plus fréquemment d'une fistule recto-urinaire. Une méconiurie et un syndrome occlusif néonatal peut être un contexte inaugural [7].

Le défect osseux sacro coccygien peut prendre des aspects très variables; il peut aller de la simple déviation latérale du coccyx jusqu′à l′agénésie du sacrum. Ces aspects sont souvent asymptomatiques et de découverte radiologique comme c’était le cas de notre malade.

La troisième composante du syndrome, qui est la masse pré sacrée, est dans la majorité des cas décrits une méningocèle antérieure. Elle peut être aussi un tératome ou l'association des deux en même temps. En effet, en 2003, il y'avait sur un total d'environ 200 cas de la triade complète de Currarino retrouvés dans la littérature, 22 patients ayant une masse pré sacrée contenant une méningocèle et un tératome [8]. Une autre série confirme que le SC peut se révéler par 4, et non pas 3, signes cliniques majeurs: anomalie sacrée typique, anus infundibulaire, masse pré-sacrée tératomateuse et/ou anomalies de la moelle terminale [2].

Enfin, et plus rarement, la masse pré-sacrée peut être une duplication digestive, un lipome, un hamartome ou un kyste dermoide [1].

L′imagerie par résonance magnétique est une modalité non invasive, sensible et spécifique du diagnostic. Elle joue un rôle important dans le diagnostic de ce syndrome. Elle pourrait être réalisée chez tout patient souffrant de constipation chronique suspect d′avoir des anomalies du tube neural et des anomalies ano-rectales. Elle permet de bien établir un bilan lésionnel qui guidera la thérapeutique [9].

Le traitement du syndrome de Currarino vise à réparer les anomalies pré sacrées en premier sous couvert d'une colostomie, réalisée dès la naissance. L′ablation complète de la masse permet d′éviter la morbidité qui y est liée et surtout de faciliter la cure de la malformation ano-rectale dans un deuxième temps. Un protocole thérapeutique proposant un calendrier rationnel des procédures chirurgicales multidisciplinaires pourrait donner une contribution valable pour le traitement du SC [5,10]. Une surveillance postopératoire à long terme s′impose dans le cadre d′une consultation pluridisciplinaire.

## Conclusion

Le syndrome de Currarino doit être évoqué devant tout élément de la triade caractéristique. Le bilan doit être digestif, médullaire et uro-génital. Il doit préciser le type des anomalies du syndrome et les malformations associées. Le diagnostic doit être écarté en cas d'anomalies constitutionnelles associées. La prise en charge précoce est essentielle pour éviter la morbidité et la mortalité de la masse pré-sacrée.

## References

[1] Arifi M, Kaddouri N, Abdelhak M, Benhmamouch MN, Barahioui M (2006). Le syndrome de Currarino: malformation anorectale, anomalie sacrée et tumeur pré-sacrée: à propos d'un cas. Gastroenterol Clin Biol..

[2] Cretolle C, Lyonnet S, Fekete C (2010). Syndrome de Currarino: Analyse clinique et génétique de 80 cas. Archives de Pédiatrie..

[3] Vargas-González R, Paniagua-Morgan F, Victoria G, de la Torre-Mondragón L, Manuel-Aparicio J (2008). Currarino syndrome: une cause rare de constipation sévère, rapport de cas et revue de la littérature. Rev Gastroenterol Mex..

[4] Saberi H, Habibi Z, Adhami A (2009). Currarino's syndrome misinterpreted as Hirschsprung's disease for 17 years: a case report. Cases J..

[5] Martucciello G, Torre M, Belloni E, Lerone M, Pini Prato A, Cama A, Jasonni V (2004). Currarino syndrome: proposal of a diagnostic and therapeutic protocol. J Pediatr Surg..

[6] Fitouri Z, Ben Slima S, Matoussi N, Aloui N, Bellagha I, Kechrid A, Ben Becher S (2007). Syndrome de Currarino: cause rare de méningites purulentes récidivantes. Med Mal Infect..

[7] Bouabdallah Y, Benhmamouch M-N, Kaddouri N et coll (2002). Le syndrome de Currarino (à propos d'un cas). Maroc médical..

[8] Thambidorai CR, Muin I, Razman J, Zulfiqar A (2003). Currarino triad with dual pathology in the presacral mass: report of a case. Dis Colon Rectum..

[9] Belkacem S, Chellaoui M, Dafiri R (2010). Le syndrome de Currarino. Feuillets de Radiologie..

[10] Crétolle C, Zérah M, Jaubert F, Sarnacki S, Révillon Y, Lyonnet S, Nihoul-Fékété C (2006). New clinical and therapeutic perspectives in Currarino syndrome (study of 29 cases). J Pediatr Surg..

